# Quantifying hope: an EU perspective of rare disease therapeutic space and market dynamics

**DOI:** 10.3389/fpubh.2025.1520467

**Published:** 2025-02-03

**Authors:** Emmanuelle Cacoub, Nathalie Barreto Lefebvre, Dimitrije Milunov, Manish Sarkar, Soham Saha

**Affiliations:** ^1^Medinsights SAS, Paris, France; ^2^ESCP Business School, Paris, France

**Keywords:** rare diseases, orphan drug designations, European Medicines Agency, market share, revenue model, pharmaceuticals, biotech, drug repurposing

## Abstract

Rare diseases, affecting millions globally, pose a significant healthcare burden despite impacting a small population. While approximately 70% of all rare diseases are genetic and often begin in childhood, diagnosis remains slow and only 5% have approved treatments. The UN emphasizes improved access to primary care (diagnostic and potentially therapeutic) for these patients and their families. Next-generation sequencing (NGS) offers hope for earlier and more accurate diagnoses, potentially leading to preventative measures and targeted therapies. In here, we explore the therapeutic landscape for rare diseases, analyzing drugs in development and those already approved by the European Medicines Agency (EMA). We differentiate between orphan drugs with market exclusivity and repurposed existing drugs, both crucial for patients. By analyzing market size, segmentation, and publicly available data, this comprehensive study aims to pave the way for improved understanding of the treatment landscape and a wider knowledge accessibility for rare disease patients.

## Introduction

Rare diseases pose a significant disease burden to patients, doctors and caregivers. They constitute a heterogeneous and conspicuous group of 7,000 indications, affecting a small number of individuals, when looked individually (< 1 in 2000) (1). However, when combined, rare diseases affect 1 in 16 people worldwide (2), leading to an estimated 300 million people being affected worldwide. Roughly 80% of the rare diseases are genetically predisposed (3), with 70% of them showing an onset during childhood years (4). However, 95% of these indications lack approved treatments, with diagnosis taking an average of 4.8 years and 30% of the pediatric population affected before the age of 5 (1). The 2021 resolution of the United Nations (UN) on rare diseases highlights the need for improved access to healthcare, especially primary care, for the affected population and their families (5). Next generation sequencing (NGS), non-coding DNA sequencing, non-invasive prenatal testing, carrier screening, advanced bioinformatics along with improvement in several other genetic screening techniques hold promise for presymptomatic and more accurate diagnosis of rare diseases (6–8). High sensitivity and specificity for diagnosing rare neurological disorders have been demonstrated previously (9). Integrated multi omics approaches have reduced the diagnosis timeline in synergy with traditional methods (10). Early diagnosis is crucial for improved health outcomes and quality of life (QoL), reducing treatment costs and easing financial burdens.

The limited market potential for rare disease drugs discourages pharmaceutical development. Policies like the 1983 US Orphan Drug Act (11) incentivize orphan drug development, leading to hundreds of approved drugs globally. Treatments like Lenmeldy (12), Hemgenix (13), and Zynteglo (14) are exorbitantly expensive, reflecting a lack of global equity to access these medications. Additionally, parents of the affected pediatric population and caregivers to face limited information, reduced treatment options, financial strain, and potential career sacrifices (15), with affected individuals often subjected to inadequate support from institutions such as schools and workplaces. Prioritizing early diagnosis, effective treatment, and increased research funding through specific policies (16) is crucial.

The European Union (EU) has taken proactive steps to address these conditions through the Orphan Drug Regulation (17), which incentivizes drug development by offering various benefits to pharmaceutical companies. Key therapeutic advancements, such as gene therapy (18), precision medicine (19), and drug repurposing (20) hold promise for improving patient outcomes. However, challenges persist, including diagnostic delays, limited treatment access, and clinical trial recruitment, particularly for ultra-rare diseases.

Recent studies have focused the understanding on rare disease therapies, at least in the EU space, to either diagnostics (1, 21) or specific therapies (22). To the best of our knowledge, our analysis is the first of its kind in providing a unique perspective and novel framework for a comprehensive evaluation of the rare diseases’ treatment landscape. Albeit concentrating only on EU and EMA approved drugs, we analyzed the scientific advancements (capturing mechanism of action diversity) and the competitive market dynamics (capturing commercial and market shares) surrounding approved and developmental therapy pipelines. This holistic approach offers a more detailed understanding of the challenges and opportunities in rare disease drug development, identifying the complexities of unmet therapeutic needs.

This study aims to offer a better understanding of the current therapeutic landscape for rare diseases in the EU by examining EMA-approved and investigational treatments in developmental pipelines. The ultimate goal of our work is to contribute to the development of innovative solutions that enhance the lives of individuals affected by rare diseases. Specifically, this study aims to reduce the cost of illness (COI) and improve the quality of life (QoL) for the patients affected with rare diseases. By achieving these objectives, we aim to empower various stakeholders such as researchers, policy makers, and pharmaceutical companies to make informed business and commercial decisions keeping in mind the current state of rare disease treatment development. Furthermore, with our market segmentation analysis we hope to facilitate the exploration of potential developmental and market opportunities within the rare disease therapeutics space.

## Methods

### Data sources

Data was extracted from publicly available information on company websites on developmental pipelines. The information on finances and market were derived from public information on company financial statements. All data sources and links are summarized in Supplementary Table S1. This study explores the therapeutic interventions’ landscape against rare diseases taking into consideration 211 drugs approved by the EMA until 2021, for rare diseases or intended for rare disease use. We also consider drugs in developmental pipeline from 43 leading biopharmaceutical companies/biotech working in the rare disease space. We have broadly classified the EMA approved drugs for rare diseases into two segments, depending on their designation status: (1) Drugs with Orphan Drug Designation (ODD) (17): Orphan drugs receive regulatory incentives and market exclusivity from the EMA due to their specific development for rare diseases. (2) Non-Orphan Drugs for Rare Diseases: Existing medications repositioned to treat rare conditions, potentially offering a faster route to patient and market access.

The revenue analysis encompasses the global market for these drugs. This comprehensive approach provides a broader understanding of the expanding landscape of these treatments both from a development and financial point of view. These systemic study takes into account publicly available data sources like: (1) Drug developmental pipelines along with financial statements of major pharmaceutical and biotechnology companies (Supplementary Tables S1, S3, S4). (2) The EMA approved drugs information until 2021 (major present pharmaceutical assets) which ensures data integrity, coverage and facilitates future research endeavors (Supplementary Tables S3–S7). The market share for rare disease therapeutics is segmented based on: (1) Pharma-Specific market shares: Examining the market position of pharmaceutical companies with these therapies. (2) Disease-Specific market shares: Understanding the market proportions of the analyzed rare diseases.

### Systematic analysis using publicly available pipeline details and financial reports

The study was performed using open-source information and data to elucidate the landscape of approved and in-development therapeutic interventions for rare diseases, taking into account the research and development (R&D) pipelines of the analyzed biopharmaceutical companies/biotech (Supplementary Table S1), predominantly in the EU. Some of the key outputs analyzed in this study include the drug name, targeted disease, the mechanism of action of the interventions, therapy areas in which these interventions fall into, collaboration status and phase of clinical development. Furthermore, the locations of the key players were analyzed to obtain a comprehensive view of the geographical space.

The second study takes into account the approved interventions in the EMA, with and without ODD status, and intended for usage in rare diseases. The annual financial reports (2023) of companies holding the market authorization of the interventions were analyzed for the product specific revenue, product specific percentage of market shares, indication specific market share distribution, company specific and disease area specific market share distribution (Supplementary Tables S5–S7).

This multifaceted data analysis of market share and corporate pipelines delineates the rare disease therapeutic landscape and elucidates market dynamics in rare diseases. This study does not encompass approved therapies and in-development assets by the Food and Drug Administration (FDA, USA) and in other regions of the world.

### Identification of interactions between indications and companies

To gain a global understanding of how a company’s developmental pipelines impact the R&D landscape for a specific rare disease and the therapeutic axes, we employed a bipartite network analysis. First, we constructed a network where companies are represented as nodes and make up the first layer of the network. These company nodes were connected to two distinct sets of nodes on the other side. The first layer containing the company nodes is then connected to two additional layers. The nodes in the second layer represent company collaborators which the companies in the first layer may work with (or that were acquired as a result of mergers and acquisitions). The nodes in the third layer represent diseases currently under development by the companies the in the first layer. In situations where a pharmaceutical company, is solely responsible for both drug discovery a direct connection between the first and third layers was made. This bipartite structure allows us to capture not only the company’s research focus but also their collaborative landscape.

We then removed individual companies from the first layer of the network, simulating a company going out of business or withdrawing from a particular research area. To assess the impact of this removal, we analyzed the interactions in the resulting network, considering two key factors. Firstly, we quantified the total number of companies remaining that would actively remain in the specific disease of interest. Secondly, we analyzed the number of unique mechanisms of action (MoA) represented by the companies. By analyzing the diversity of MoA, we assessed the potential impact on the range of therapeutic approaches being explored for the disease. This combined measure offered a comprehensive understanding of how a company’s developmental pipelines affect both the overall research effort and the richness of approaches (MoA) for a particular disease (Appendix 3).

### Drug-protein interaction network

Drug-protein interaction networks were constructed using the STITCH database (version 5.0) (23). This database integrates experimental and curated data with text mining and prediction algorithms, resulting in a comprehensive network of chemical-protein interactions. For each drug of interest, both ODD and non-ODD, the corresponding STITCH network was retrieved and visualized. The strength of the drug-protein interactions in STITCH is quantified by a confidence score ranging from 0 to 1, where higher scores indicate stronger evidence for the interaction. To identify the impacted pathways, we extracted the proteins involved in the drug-protein interactions and mapped them to known biological pathways using pathway analysis tools such as Reactome or KEGG. Finally, the drugs with top 30 MoAs were counted and presented in the form of a bar graph.

### Visualizations

A scatter plot visualized the relationships between various parameters, including market share, revenue of top-selling assets, competitive index, and collaborative index. The data was aggregated and collected in Microsoft Excel, using Excel functions such as *vlookup*. The plotting was done using Prism v10.2.3 (GraphPad) and custom-made scripts using Python programming language. Figure panels were prepared in affinity designer and Inkscape softwares.

## Results

### Global map of pharmaceutical players, therapy areas of interventions and their corresponding mechanisms of action

**Figure 1:**
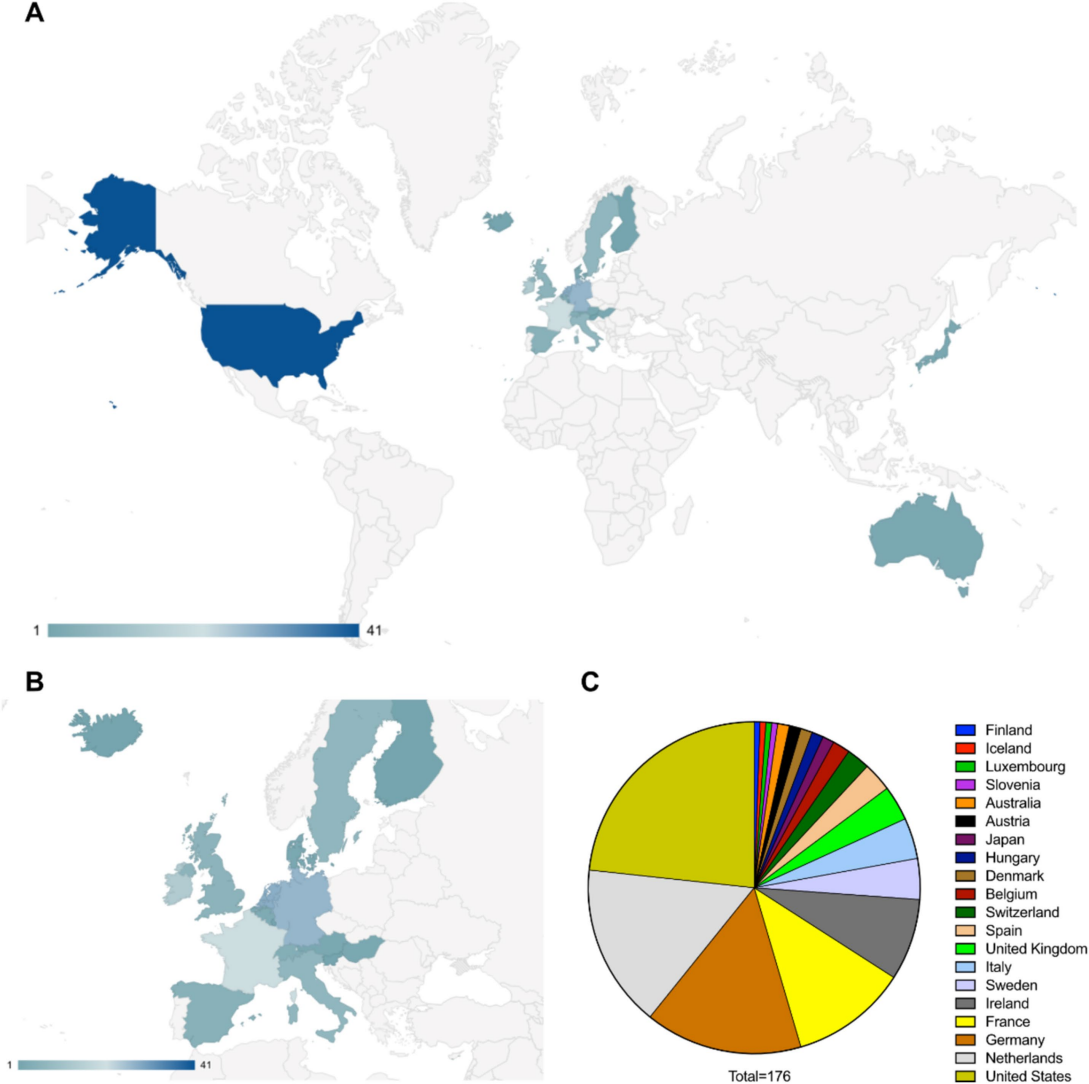
**(A)** Worldwide distribution of pharmaceutical entities having approved and in development therapeutics against rare diseases (data considered from EMA specific documents, companies from other regions in the world may not be covered in this study). **(B)** European distribution of pharmaceutical entities having approved and in development therapeutics against rare diseases. **(C)** Distribution of countries according to the number of pharmaceutical entities headquartered there.

The global distribution shows that the pharmaceutical entities involved in the development and approval of drugs for rare diseases are headquartered mostly in the developed countries. The United States is the leading location for the headquarters followed by the EU nations, Japan and Australia (Figures 1A,B). Some of the leading countries hosting these headquarters are the Netherlands, Germany, France, Ireland, Sweden, Italy, United Kingdom, Spain, Switzerland and Belgium (Figure 1C).

**Figure 2:**
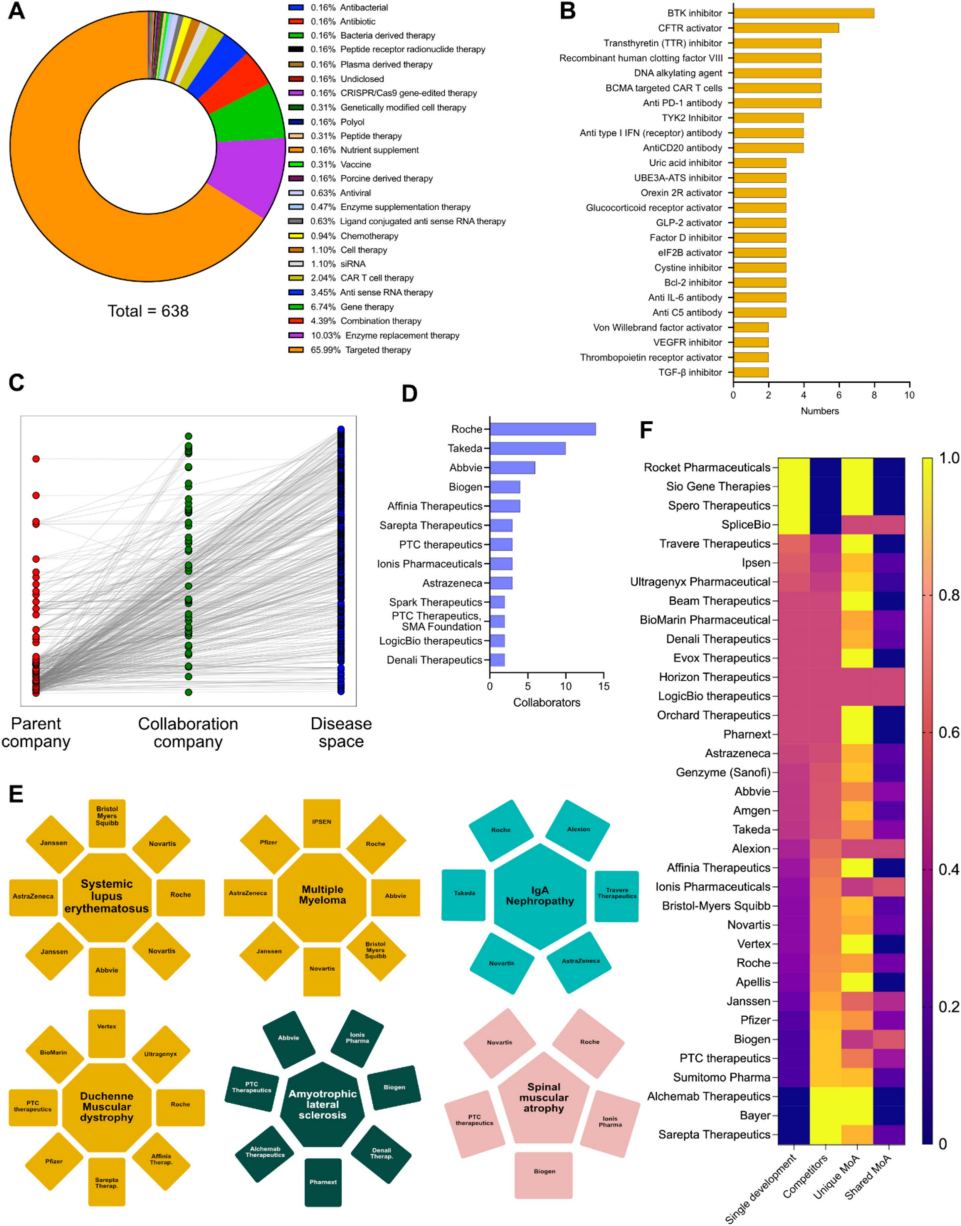
**(A)** Intervention specific therapy area distribution in the therapeutic landscape. **(B)** Intervention specific mechanism of action (MoA) distribution of the therapeutic landscape. **(C)** Connectivity matrix connecting the parent company, collaborative companies and the diseases targeted in their developmental pipeline. **(D)** Top pharmaceutical companies having the highest number of collaborations in their developmental pipelines. **(E)** Top 6 disease areas dominated by multiple pharmaceutical players increasing competitiveness. **(F)** Heatmap showing the innovative index and competitive index (disease and MoA) of different pharmaceutical players having developmental pipelines in rare disease space (Yellow: Highest; Blue: Lowest).

Key therapies for rare diseases include targeted therapy, ERT, gene therapy, combinatorial therapy, antisense RNA therapy, CAR-T cell therapy, and siRNA-based interventions (Figure 2A). The mechanism of action landscape encompasses enzyme Inhibitors (such as BTK, TYK2, TTR, UBE3A-ATS, Bcl-2, VEGFR inhibitors and others), activation therapies (such as CFTR, orexin 2 receptor and GLP-2R activators), antibody-based therapies (such as anti-PD-1, anti-type I interferon receptors, anti-CD20, anti-IL-6 and anti-complement system C5), Alternative therapeutic strategies such as recombinant human clotting factor VIII, DNA alkylating agents, CAR T-cell therapy targeting B-cell maturation antigen, and drugs that modulate cellular processes like protein translation (elF2B activation) and fibrosis (TGF-*β* inhibition) are also implicated (Figure 2B). In the course of this study, 641 rare disease interventions, 530 unique MoAs and 25 unique modalities were analyzed, along with individual pipeline analyses for the major players in rare diseases (Supplementary Figures S1, S2). Connectivity across parent companies sponsoring the development of an intervention, their collaboration and partnering with other companies/biotech and the targeted disease shows that collaborations are a key component of drug development in rare disease therapies (Figure 2C). Hoffmann-La Roche AG, Takeda, AbbVie, Biogen, Affinia therapeutics, Sarepta therapeutics, PTC therapeutics, Ionis pharmaceuticals, AstraZeneca AB, Spark therapeutics, LogicBio therapeutics and Denali therapeutics take the lead in partnerships and collaborations in developmental pipelines (Figure 2D), in key therapeutic areas such as systemic lupus erythematosus [Novartis, Hoffmann-La Roche AG, AbbVie, Janssen, AstraZeneca and Bristol-Myers Squibb (including Celgene)], multiple myeloma (Ipsen Pharmaceuticals, Hoffmann-La Roche AG, AbbVie, Novartis, Janssen, AstraZeneca, Pfizer and Bristol-Myers Squibb), Duchenne muscular dystrophy (Hoffmann-La Roche AG, Vertex Pharmaceuticals, Ultragenyx, Affinia therapeutics, Sarepta therapeutics, Pfizer, PTC Therapeutics and BioMarin International), amyotrophic lateral sclerosis (Biogen, Ionis Pharmaceuticals, AbbVie, PTC therapeutics, Alchemab therapeutics, Pharmanext and Denali therapeutics), IgA nephropathy (Hoffmann-La Roche AG, Alexion (part of AstraZeneca), Travere therapeutics, AstraZeneca AB, Novartis and Takeda) and spinal muscular atrophy (Hoffmann-La Roche AG, Novartis, Biogen, Ionis Pharmaceuticals and PTC therapeutics). All these players have either single or multiple programs targeting the indications. In the case of multiple programs, the corresponding pharmaceutical companies are represented by multiple occurrences (Figure 2E). The competitive landscape of drug development for rare diseases was further granulated using:

(i) Single development: Only entity developing intervention against specific indications;
(ii) Competitors: Multiple entities involved in therapeutic interventions development against the indication;
(iii) Unique mechanism of action (MoA): Number of unique therapeutic axes used for therapeutic development from a mechanistic point of view; and
(iv) Shared MoAs: Number of shared therapeutic axes employed for therapeutic development from a mechanistic point of view.

We were able to conclude that Rocket pharmaceuticals, Sio Gene therapies, Sphero therapeutics and SpliceBio have a high degree of innovative index, but very low degree of competitive index. Companies like Sarepta Therapeutics, Bayer AG, Alchemy Therapeutics, Sumitomo Pharmaceuticals, Janssen, Biogen, Hoffmann-La Roche AG, PTC therapeutics, Vertex Pharmaceuticals, Ionis Pharmaceuticals, Affinia Therapeutics, Alexion (part of AstraZeneca AB) have a low degree of innovative index and a very high degree of competitive index, while AbbVie, Amgen, Genzyme (part of Sanofi), AstraZeneca AB, Orchard therapeutics, LogicBio Therapeutics have a comparable innovative and competitive index (Figure 2F). Furthermore, analyses of all the companies involved in therapeutic development in unique axes of MoAs yields biotech entities with high degree of innovative index or competitive index (either from disease perspective or MoA perspective) and are potentially key determinants for M&A activities (Figure 2F).

### Global distribution of EMA approved drugs (until 2021) with and without ODD status against rare indications having market shares

**Figure 3:**
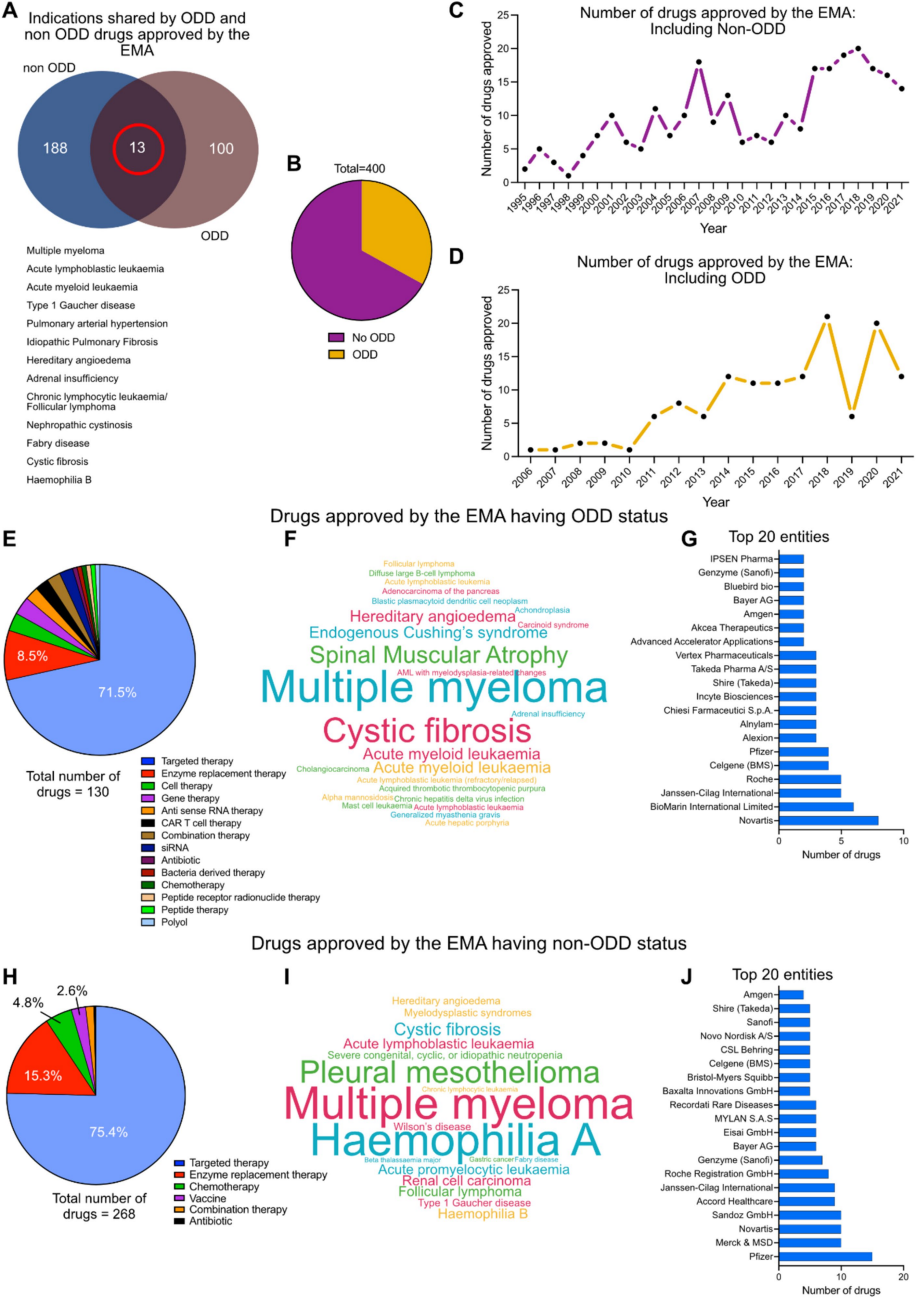
**(A)** Indications shared by EMA approved drugs for rare diseases with and without ODD. **(B)** Distribution of total number of EMA approved drugs having ODD and no ODD. **(C)** Approval timeline of drugs against rare diseases by EMA without ODD (1995–2021). **(D)** Approval timeline of drugs against rare diseases by EMA with ODD (2005–2021). **(E)** Therapy area distribution of drugs with ODD (*n* = 130). **(F)** Cloud diagram showing the distribution of rare indications against which the ODD designated drugs were being approved. **(G)** Top 20 pharmaceutical entities involved in this intervention space with ODD. **(H)** Therapy area distribution of drugs without ODD (*n* = 268). **(I)** Cloud diagram showing the distribution of rare indications against which the non-ODD drugs were being approved. **(J)** Top 20 pharmaceutical entities involved in this intervention space without ODD.

The EMA has approved interventions against 113 indications with ODD and 201 indications without ODD status till 2021.13 of these indications (multiple myeloma, acute lymphoblastic leukemia, type 1 Gaucher disease, pulmonary arterial hypertension, idiopathic pulmonary fibrosis, hereditary angioedema, adrenal insufficiency, chronic lymphocytic leukemia, follicular lymphoma, nephropathic cystinosis, Fabry disease, cystic fibrosis and hemophilia B) have known interventions, with both ODD and non-ODD status (Figure 3A). Out of the 400 interventions approved against rare diseases by EMA (Table 1), 35% have ODD status while 65% do not have ODD status (Figure 3B). There has been a continuous increase in the number of drug approvals against rare disease with and without ODD status in the last few decades (Figures 3C,D). The EMA approved drugs having ODD against rare diseases are dominated by targeted therapy (71.5%) and enzyme replacement therapy (8.5%) (Figure 3E). Some of the key indications targeted by ODD drugs are multiple myeloma, cystic fibrosis, spinal muscular atrophy, endogenous Cushing syndrome, hereditary angioedema and acute myeloid leukemia (Figure 3F). Novartis, BioMarin International Limited, Janssen Cilag International, Hoffmann-La Roche AG, Celgene (acquired by Bristol-Myers Squibb), Pfizer, Alexion (AstraZeneca partner), Alnylam pharmaceuticals, Chiesi Pharmaceutical, Incyte Biosciences, Shire (part of Takeda), Takeda pharmaceuticals, Vertex pharmaceuticals, Advanced Accelerator Applications, Akcea Therapeutics, Amgen, Bayer AG, Bluebird bio, Genzyme (part of Sanofi) and Ipsen Pharmaceuticals are the key pharmaceutical players in this group (Figure 3G).

**Table 1:** Summary of key numbers analyzed in the study.

Criteria		Numbers
Number of rare diseases studied *		295
Total number of unique drugs identified in rare diseases		611
Approved therapies by EMA (till 2021)		211
Total number of therapies in development (in development pipelines)	Early clinical (including Early Phase 1, Phase 1 and 2)	200
	Late clinical (including Phase 3 and onwards)	200
Number of unique mechanisms of actions identified *		283
Total number of pharmaceutical companies		43
Number of specific targets of approved therapeutic interventions		400
Number of drugs approved by EMA with ODD status		140
Number of drugs approved by EMA with non-ODD status		268

*These numbers are not presented in the main text, and are calculated from Supplementary information.

The EMA approved drugs against rare diseases without ODD status are dominated by targeted therapy (75.4%), enzyme replacement therapy (15.3%) and chemotherapy (4.8%) (Figure 3H). Some of the key indications targeted by non-ODD drugs are multiple myeloma, hemophilia A, pleural mesothelioma, cystic fibrosis and acute lymphoblastic leukemia (Figure 3I). Pfizer, Merck, Novartis, Sandoz, Accord healthcare, Janssen International, Hoffmann-La Roche AG, Genzyme (part of Sanofi), Bayer AG, Eisai Gmbh, Mylan SAS, Recordati Rare Diseases, Baxalta Innovations, Bristol Myers Squibb, Celgene (acquired by Bristol-Myers Squibb), CSL Behring, Novo Nordisk, Sanofi, Shire (part of Takeda) and Amgen remain key players in the non-ODD group (Figure 3J).

### Chemical network and mechanism of action driven landscape analysis of approved drugs against rare indications with and without ODD designation

**Figure 4:**
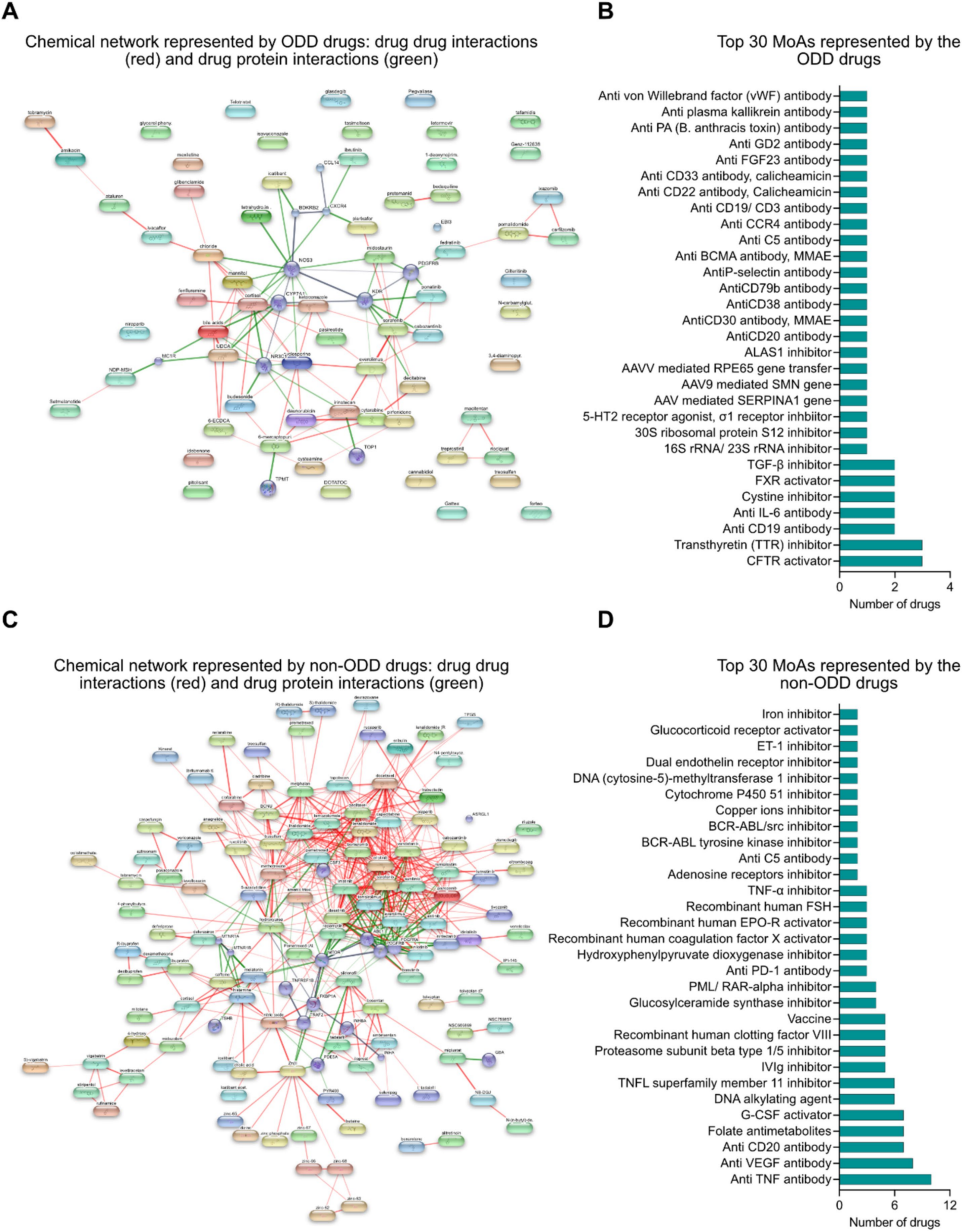
**(A)** Chemical network represented by ODD designated interventions: drug–drug (red) and drug-protein (green) interaction framework. **(B)** Top 30 mechanisms of actions represented by interventions with ODD designation. **(C)** Chemical network represented by non-ODD interventions: drug–drug (red) and drug-protein (green) interaction framework. **(D)** Top 30 mechanisms of actions represented by interventions without ODD designation.

Drug–drug (red lines) and drug protein (green lines) interaction networks represent the chemical landscape of the interventions. It shows that the drugs are quite sparse spatially which is due to the presence of innovative interventions targeting different biomarkers in the rare disease space which has received ODD (Figure 4A). Some of the key mechanisms of action represented by the ODD designated drugs are small molecule targeted therapy (such as CFTR activator, transthyretin (TTR) inhibitor, cysteine inhibitor, FXR inhibitor, TGF-*β* inhibitor, 16S/23S rRNA inhibitor, 30S ribosomal protein S12 inhibitor, 5-HT2 receptor agonist, σ1 receptor inhibitor, ALAS1 inhibitor), antibody-mediated targeted therapy (such as anti CD19 antibody, anti IL6 antibody, anti C5 antibody, anti CCR4 antibody, anti CD19/CD3 bispecific antibody, anti FGF23 antibody, anti CD20 antibody, anti GD2 antibody, anti-PA (*B. anthracis* toxin) antibody, anti-plasma kallikrein antibody, anti von Willebrand factor (vWF) antibody), antibody-drug conjugate (such as anti CD30 antibody/monomethyl auristatin E (MMAE), anti CD22 antibody/Calicheamicin, anti CD33 antibody/Calicheamicin) and gene therapy (such as AAV mediated SERPINA1 gene transfer, AAV9 mediated SMN gene transfer, AAV mediated RPE65 gene transfer) (Figure 4B).

Similarly, drug–drug (red lines) and drug-protein (green lines) interaction networks among the non-ODD interventions might suggest that these interventions are mostly repositioned from other non-rare indications that share common targets and mechanisms of action (Figure 4C). The key mechanisms of action represented by the non-ODD designated drugs are small molecule targeted therapy, antibody mediated targeted therapy, enzyme replacement therapy and alternative therapies like DNA alkylating agent and folate antimetabolites (Figure 4D).

### Market share landscape of therapeutic space against rare diseases with and without ODD status

**Figure 5:**
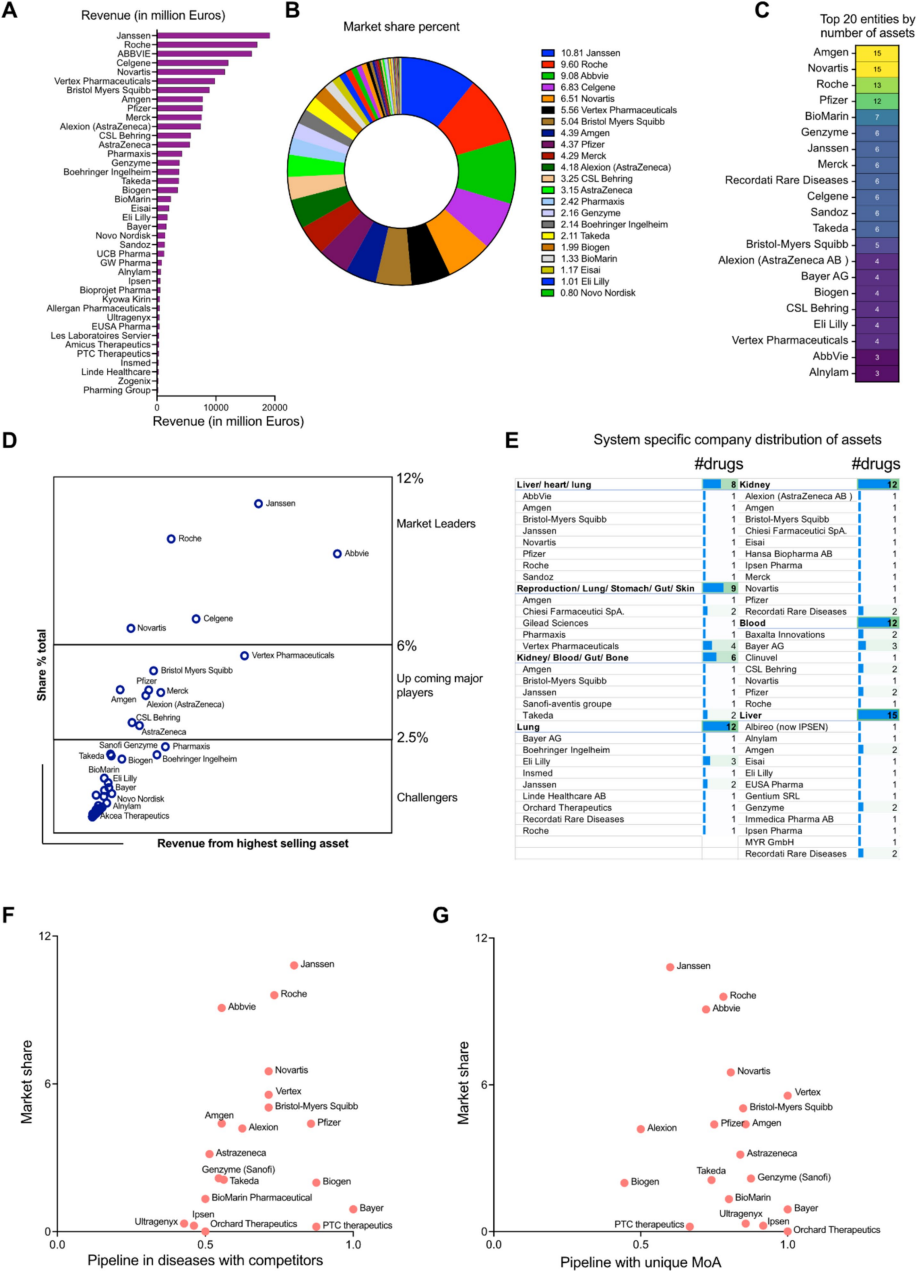
**(A)** Revenue distribution among the pharmaceutical entities (in million dollars) in rare disease space. **(B)** Rare disease therapeutic market share distribution among the pharmaceutical entities (percentage). **(C)** Top 20 pharmaceutical companies having the highest number of money-making assets. **(D)** Market leader estimation using the corresponding correlation coefficients between the total revenue and highest grossing asset. **(E)** Physiological system specific company distribution of assets. **(F)** Scatter plot showing the competitive index for disease space targeted by the pharmaceutical players (developmental pipelines) in perspective to the total market shares (approved drugs). **(G)** Scatter plot showing the innovative index driven by unique MoAs targeted by the pharmaceutical players (developmental pipelines) in perspective to the total market shares (approved drugs).

A review of the pharmaceutical industry identified 20 key players by revenue and market share specific to rare diseases. Janssen (10.8%) came first in the list, followed by Hoffmann-La Roche (9.6%) and AbbVie (9.1%). Mergers and acquisitions are reflected, with Celgene (6.8%) included under Bristol-Myers Squibb and Alexion (4.2%) under AstraZeneca. The remaining companies include Vertex Pharmaceuticals (5.5%), Novartis (6.5%), Amgen (4.4%), Pfizer (4.4%), Merck (4.3%), CSL Behring (3.3%), Pharmaxis (2.4%), Genzyme (part of Sanofi, 2.2%), Boehringer Ingelheim (2.1%), Takeda (2.1%), Biogen (2.0%), BioMarin (1.3%), Eisai (1.1%), and Eli Lilly (1.0%) (Figures 5A,B). In addition, Amgen, Novartis, Hoffmann-La Roche AG, Pfizer, BioMarin international, Celgene (part of Bristol-Myers Squibb) Genzyme (part of Sanofi), Janssen, Merck, Recordati Rare Diseases, Sandoz, Takeda, Bristol-Myers Squibb, Alexion (part of AstraZeneca), Bayer AG, Biogen, CSL Behring, Eli Lilly, Vertex Pharmaceuticals AbbVie and Alnylam Pharmaceuticals also boast a large pharmaceutical portfolio of assets (Figure 5C). The market leaders of this therapeutic space were estimated using a correlation study between the total revenue and the highest grossing asset of each of the companies. It showed that there are three separate divisions among the players: The market leaders (6–12%): Janssen, AbbVie, Hoffmann-La Roche AG and Novartis and Celgene (part of Bristol-Myers Squibb); The upcoming major players (2.5–6%): Vertex pharmaceuticals, Bristol-Myers Squibb non-Celgene portfolio, Pfizer, Amgen, Merck, Alexion (part of AstraZeneca), CSL Behring and AstraZeneca AB non-Alexion portfolio; The challengers to the market space (<2.5%): Pharmaxis, Boehringer Ingelheim, Takeda, Biogen, Genzyme (part of Sanofi), Bayer AG and Alnylam Pharmaceuticals among others (Figure 5D). Physiological system specific company distribution of assets was analyzed and it provides the granularity of the number of interventions in correlation to the body system their targeted indications affect. A further analysis by organ system unveiled a diverse distribution of interventions. Several companies, like AbbVie, Amgen, Bristol-Myers Squibb, Janssen, Novartis, Pfizer, Roche, and Sandoz focus their efforts with a single intervention specific to the complex liver/heart/lung or the broader reproduction/lung/stomach/gut/skin systems. Similarly, companies like Amgen, Bristol-Myers Squibb, Janssen, and Sanofi hold a single intervention in the kidney/blood/gut/bone space, suggesting a more targeted approach in these areas. However, the landscape shifts when examining companies with multiple interventions within specific organ systems, where we observe a concentrated focus on those specific systems. For example, Janssen and Eli Lilly take a leading role in the lung system with two and three interventions, respectively. Similarly, Bayer AG, Recordati Rare Diseases, and Amgen (among others) hold multiple assets in the blood and liver systems, indicating a strategic investment in these critical areas. This trend highlights the variety of approaches companies carry out when developing interventions, with some opting for a broader reach and others focusing on specific organ systems with multiple offerings (Figure 5E).

We defined the competitive index of a company based on quantitative assessment of the existing competitors in the therapeutic space. If a therapeutic strategy had more than one player, it was deemed as competitive. On the other hand, if the mechanism of action of the therapeutic asset was unique to a particular company, it could indicate high innovation index. A scatter plot between the total market share of the pharmaceutical entities in rare therapeutics space and their total assets in pipeline shared by other competitors gives an estimate of the market leaders in two perspectives: Approved drugs in market and drug development in a competitive environment (Figure 5D). Janssen, Hoffmann-La Roche AG and Novartis are the leaders having the highest market shares and richest developmental pipeline in the competitive disease environment (Competitive index closer to 1). Market leaders like AbbVie have a high share of the market space but play in a lower risk environment (Competitive index close to 0.5). The upcoming players like Vertex Pharmaceuticals, Bristol Myers Squibb (including Celgene developmental portfolio), Pfizer and Alexion (part of AstraZeneca AB) have median market shares and also are a part of a highly competitive disease landscape (Competitive index closer to 1). Amgen and AstraZeneca (without Alexion portfolio) have median market percentages but target diseases which are shared to a lesser extent among the competitors (Competitive index closer to 0.5). Biogen, Bayer AG and PTC therapeutics are in a strong competitive position (Competitive index closer to 1), but have comparatively less market shares than its competitors. Entities like Genzyme (part of Sanofi), Takeda, BioMarin Pharmaceuticals, Ipsen Pharmaceuticals, Ultragenyx and Orchard therapeutics are working in therapeutic areas which can accommodate higher levels of competition (Competitive index closer to 0.5) and despite their market shares being lower, they have the opportunity to expand their market positioning (Figure 5F). Analyzing the unique mechanism of action of the interventions in the developmental pipeline targeting rare diseases, all the prominent market players like Vertex Pharmaceuticals, Hoffmann-La Roche AG, AbbVie, Takeda Novartis, Bristol-Myers Squibb (including Celgene portfolio), Amgen, Pfizer, AstraZeneca AB, Genzyme, BioMarin International have a very high innovation index (close to 1) and are distributed across the entire market share landscape (high to low). Entities like Janssen, Alexion (part of AstraZeneca AB), Biogen, PTC Therapeutics cover are distributed in the market share landscape having relatively lower innovation index (close to 0.5) (Figure 5G).

### Market share landscape of disease and system specific therapeutics against rare diseases

**Figure 6:**
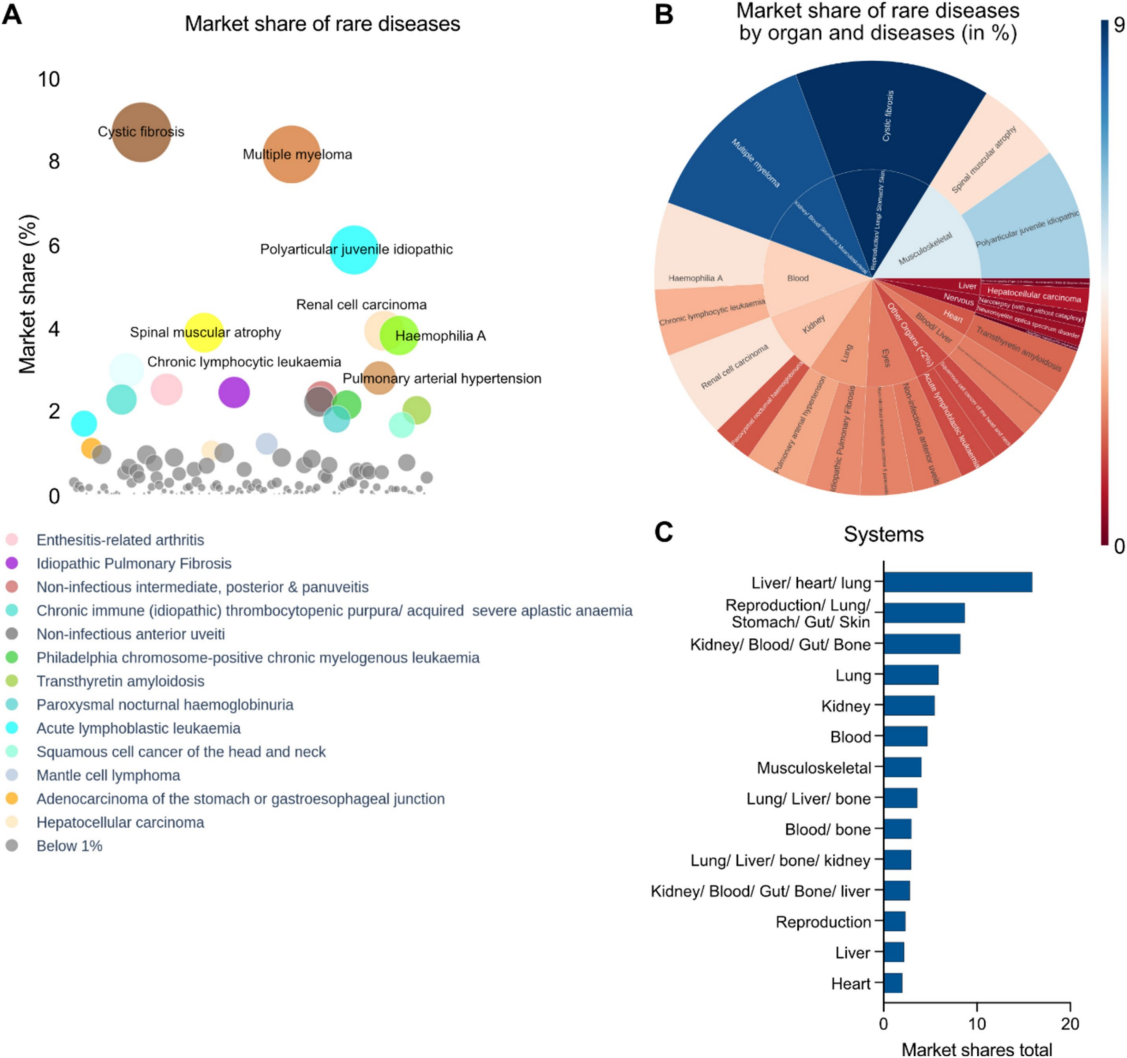
**(A)** Disease specific market share analysis of approved interventions (EMA) in rare disease therapeutics space. **(B)** Radial map showing disease and organ specific market share analysis (Blue: Highest; Brown: Lowest) of approved rare disease therapeutics in the EMA. **(C)** Top disease specific organ systems targeted by EMA approved drugs in context of market shares.

An analysis of disease specific market share was performed taking into account the total revenues of multiple drugs against the indications. We implemented a bubble diagram to visualize the relationships which shows the top indications with the highest market share given their approved therapies: cystic fibrosis, multiple myeloma, polyarticular juvenile idiopathic arthritis, renal cell carcinoma, hemophilia A, spinal muscular atrophy and chronic lymphocytic leukemia (Figure 6A). A physiological system (single or multi system) specific market share analysis takes into account the total cash flow from interventions targeting the diseases affecting the specific systems which was represented by a radial map (Figure 6B). The top organ systems having the highest pharmaceutical assets are in multiple organs encompassing the liver, heart, lung, stomach, gut, skin and others such as the kidney, blood, bone, musculoskeletal systems, or affecting distinct systems such as reproduction, liver and heart, among others (Figure 6C). It should be noted that there might be overlap in organ system assignments. For example, both “liver/heart/lung” and “lung/liver/bone” include “lung” and “liver.” This suggests that the assets were evaluated individually in each organ system, and the distinct assignments reflect the unique combinations of effects identified in each case (Figure 6C).

According to our cumulative analysis, interventions against rare diseases approved by the EMA have a global market of around 170 billion US dollars as of the fiscal year 2022–2023. There are around 611 unique interventions that were analyzed in this study, which consists of 211 approved interventions by EMA and 400 interventions that are in developmental pipelines of 43 different pharmaceutical players. The leaders of the market are Janssen Pharmaceuticals, AbbVie, Hoffmann-La Roche AG, Bristol-Myers Squibb, Vertex Pharmaceuticals and Novartis and this estimate aligns with the 2028 estimate of the leading pharmaceutical companies worldwide by projected orphan drug sales share (24).

## Discussion

People with rare diseases face significant challenges due to limited treatment options and a lack of scientific awareness, often leading to delayed diagnoses, inadequate care, and diminished quality of life. Our study reveals the existing approved and in-development pipelines of interventions in the EU pharmaceutical space. Targeted therapies, as demonstrated by the existing drug pipelines across biotech and pharma, are emerging as the most dominant approach. These therapies are precise, honing in the specific biological pathways that underlie rare diseases (25). Targeted therapies, such as enzyme replacement and gene therapy, offer more precise and potentially curative treatments for rare diseases compared to traditional medications. Enzyme replacement therapies address enzyme deficiencies (26), while gene therapy tackles the root cause of certain genetic conditions by introducing healthy genes (27). This shift toward precision medicine represents a significant leap forward in the treatment of rare diseases (28).

Our analysis reveals a diverse commercial landscape in the rare disease drug development space. While companies like Rocket Pharmaceuticals and Sio Gene Therapies exhibit high innovation but face low competition, established players like Bayer AG, Janssen, Biogen, Hoffmann-La Roche AG operate in more competitive environments, highlighted by assets sharing common mechanisms of action. On the other hand, AbbVie, Amgen and AstraZeneca demonstrate a more balanced approach. Biotech entities pioneering unique MoAs or targeting novel disease areas are highly attractive from M&A perspective, emphasizing the importance of both innovation and competitive positioning in this dynamic market. Our study indicates a significant increase in EMA-approved drugs for rare diseases, with both ODD-designated and non-ODD-designated therapies (17). Targeted therapies dominate both categories, while enzyme replacement therapies and other modalities are also prevalent. These findings underscore the growing importance of both ODD and non-ODD approaches in addressing the diverse needs of patients with rare diseases. The ODD drugs form a more distinct network of interactions with their target proteins, suggesting they likely target unique biological pathways. This highlights the cutting-edge nature of ODD drugs –to discover “best-in-class” or “first-in-class” therapies. The concept of a drug-protein network provides a compelling way to visualize the intricate interactions between medications and the protein targets. The stronger the interaction between a drug and a protein, the thicker the connecting line (23). The connection strength is dependent on the confidence or relevance of the reported interaction. STITCH generates a network by integrating interactions from diverse sources, including text mining and PubChem. A confidence score is assigned to each chemical-protein pair, quantifying the likelihood of the interaction based on the underlying evidence (29). In the case of ODD drugs, these networks appear more discrete, showcasing their unique mechanisms while for non-ODD drugs, the network is denser and more connected, reflecting the life cycle management (LCM) and drug repositioning opportunities (20).

While scientific innovation holds the key to unlocking new treatment options, the commercial landscape plays a critical role in ensuring these therapies reach the patients who need them the most. This study delves deeper into the market share percentages, revealing the pharmaceutical companies who are leading the generated revenue. Companies like Janssen, Roche, AbbVie, Bristol-Myers Squibb and Novartis are at the forefront, demonstrating their market reach and commercial ability in addressing rare disease research and development. It is evident from the analysis of the rare disease therapeutics market that the dynamic landscape is dominated by a few key players, including Janssen, Hoffmann-La Roche, and AbbVie. While market share is crucial, the analysis highlights the importance of a diversified drug portfolio, with a focus on both high-market-share indications and innovative approaches. Companies like Vertex and Bristol-Myers Squibb are navigating a balance between market leadership and a competitive development environment, as evidenced by their presence in both high-market-share segments and competitive disease landscapes. Bayer AG is a new entrant in this market space focusing on drug pipeline expansion of therapeutic portfolio. The analysis also highlights the strategic importance of organ system focus. Some biopharmaceutical companies, like Janssen and Eli Lilly, prioritize specific systems, such as the lung, while others maintain a broader approach involving multiple systems in their developmental pipeline. An example for a broad approach, according to our analysis is Amgen, which focuses on blood and liver systems. This suggests that pharmaceutical companies often opt for a tailored strategy, balancing both market potential and internal strengths in rare disease drug development. Furthermore, the analysis emphasizes that the Biopharmaceutical companies with high innovation index, driven by unique mechanisms of action, are well-positioned for future growth, regardless of their current market share. This is evident in the analysis of biopharmaceutical companies like Vertex and Hoffmann-La Roche AG, which demonstrate strong presence across the market share landscape while maintaining high innovation indices. Indeed, the innovation index proposed here only relies on unique mechanisms of action of assets exclusive to the pharmaceutical company (i.e., the mechanisms of action is not observed to repeat across other companies), but this highlights the need for continued investment in research and development to discover novel therapies and expand the treatment options available for patients with rare diseases. The increasing number of players also suggests a growing awareness of the importance of this field. New developmental possibilities lie in precision medicine (30), where targeted therapies are even more granulated (31), with the vision of tailoring therapies to the unique genetic fingerprint of each patient or patient groups (32). Additionally, gene therapy advancements with technologies such as CRISPR (33–35), AAV-mediated therapies (36–38), and antisense oligonucleotide approaches (39, 40) offer the potential for disease-rectifying cures through precise genetic code editing. As described in this study, we can identify hidden patterns and connections, accelerate drug discovery and improve our understanding of rare diseases by harnessing the power of big data analytics (41). Collaborative efforts between different stakeholders are crucial to bridge the gap in rare disease research. Philanthropic funding plays a vital role in supporting this progress and ensuring that everyone has equitable access. Foundations dedicated to rare diseases (42–44) and private philanthropists (45) try to address the gap between the available therapies and the small market size associated with rare diseases.

Patient associations play a crucial role in raising awareness about rare diseases (46, 47), advocating for patient needs, and driving research and funding initiatives. By educating the public and policymakers, they help address the high unmet needs associated with these conditions and accelerate the development of new treatments (48). Patient associations facilitate clinical trials for rare diseases by connecting researchers with specific patient populations (49, 50).

Our work provides a comprehensive framework to assess competitive landscape to identify potential partnerships, licensing opportunities, or areas where differentiation is possible. It also provides strategic insights to guide investment decisions and resource allocation, clinical trial design and optimization, reimbursement landscapes, and regulatory hurdles to optimize commercialization strategies. This study has two potential limitations. First, as it is an EU perspective of the rare disease therapeutics landscape, it includes only the EMA approved drugs and not the FDA approvals. However, the expanded intervention landscape will be studied in the future. Second, some approved interventions have multifaceted disease targets, both rare and non-rare. This might have led to some overlapping market shares from non-rare diseases being incorporated in the total market size estimation for the EMA approved rare disease therapeutics.

## Data Availability

The original contributions presented in the study are included in the article/Supplementary material, further inquiries can be directed to the corresponding authors.

## Supplementary material

Supplementary Figure S1 Pipeline of assets in leading pharmaceutical companies such as Amgen, Novartis, Roche, Pfizer, and IPSEN.

Supplementary Figure S2 Pipeline of assets in leading pharmaceutical companies such as Biogen, AstraZeneca/ Alexion, AbbVie, Takeda, and Janssen.

Supplementary Table S1 List of data sources used in the report.

Supplementary Table S2 Distribution of pharmaceutical players and biotech across the world.

Supplementary Table S3 List of drugs and interventions analyzed in the present study.

Supplementary Table S4 List of drugs, MoA and linked pharmaceutical companies.

Supplementary Table S5 Landscape of rare disease therapeutics having non-orphan drug designation.

Supplementary Table S6 Landscape of rare disease therapeutics having orphan drug designation.

Supplementary Table S7 List of developmental pipelines across pharma companies with the largest market shares in rare diseases.

## Appendix

Appendix 1 Landscape of rare disease therapeutics having orphan drug designation.

Appendix 2 Landscape of rare disease therapeutics having non-orphan drug designation.

Appendix 3 List of drugs in development by companies and segregated by indications and MoA.

Appendix 4 Revenue and market share landscape for the interventions analyzed.
