# Supplementary figures and images for: Quantifying hope: an EU perspective of rare disease therapeutic space and market dynamics
Source: Front Public Health. 2025 Feb 3;13:1520467. doi: 10.3389/fpubh.2025.1520467 (PMC11830808; doi:10.3389/fpubh.2025.1520467)

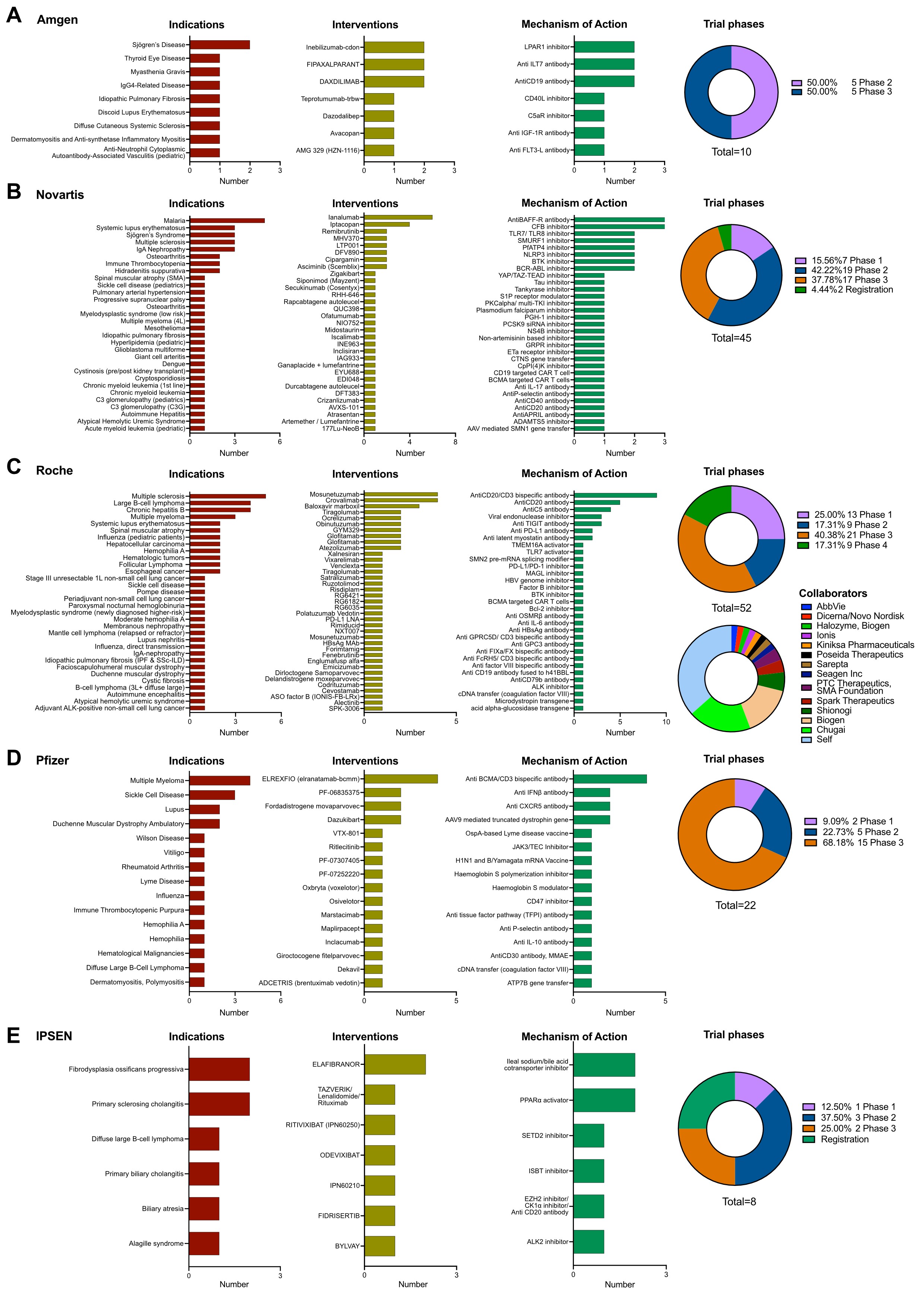

Supplement: Supplementary Figure S1 — Pipeline of assets in leading pharmaceutical companies such as Amgen, Novartis, Roche, Pfizer, and IPSEN. [file Image_1.JPEG]

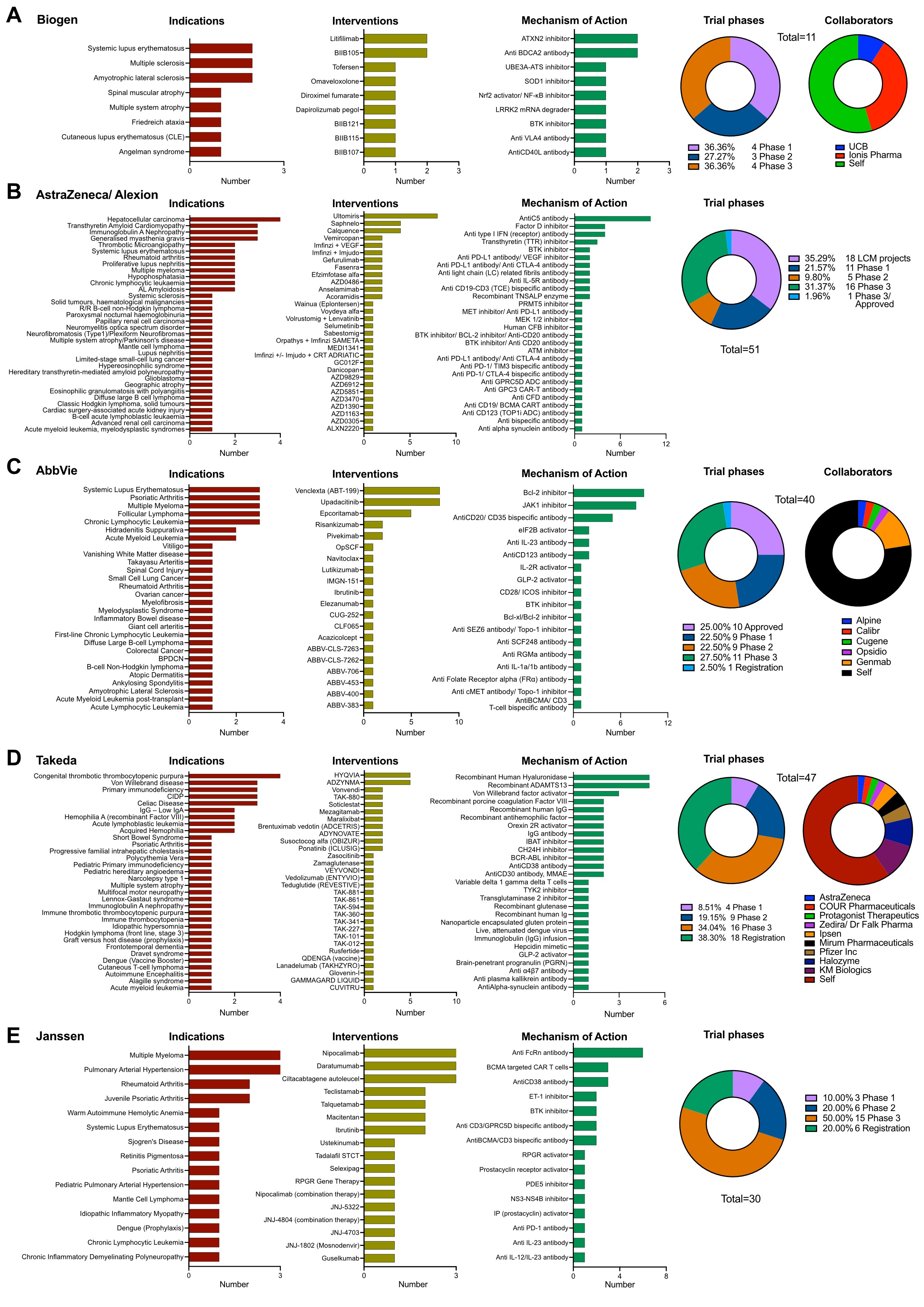

Supplement: Supplementary Figure S2 — Pipeline of assets in leading pharmaceutical companies such as Biogen, AstraZeneca/ Alexion, AbbVie, Takeda, and Janssen. [file Image_2.JPEG]
